# Cyclic Helix B Peptide Prolongs Skin Allograft Survival *via* Inhibition of B Cell Immune Responses in a Murine Model

**DOI:** 10.3389/fimmu.2021.682749

**Published:** 2021-05-12

**Authors:** Long Zheng, Xuanchuan Wang, Linkun Hu, Wenjun Gao, Weitao Zhang, Xuepeng Zhang, Chao Hu, Ruiming Rong, Cheng Yang, Dong Zhu

**Affiliations:** ^1^ Department of Urology, Zhongshan Hospital, Fudan University, Shanghai, China; ^2^ Shanghai Key Laboratory of Organ Transplantation, Shanghai, China; ^3^ Department of Urology, The First Affiliated Hospital of Soochow University, Suzhou, China; ^4^ Department of Critical Care Medicine, Zhongshan Hospital, Fudan University, Shanghai, China; ^5^ Department of Blood Transfusion, Zhongshan Hospital, Fudan University, Shanghai, China; ^6^ Zhangjiang Institute of Fudan University, Shanghai, China

**Keywords:** cyclic helix B peptide, antibody-mediated rejection, plasma cells, germinal center B cells, Tfh cells, donor specific antibodies

## Abstract

Antibody-mediated rejection (AMR) represents a major cause of allograft dysfunction and results in allograft failure in solid organ transplantation. Cyclic helix B peptide (CHBP) is a novel erythropoietin-derived peptide that ameliorated renal allograft rejection in a renal transplantation model. However, its effect on AMR remains unknown. This study aimed to investigate the effect of CHBP on AMR using a secondary allogeneic skin transplantation model, which was created by transplanting skin from BALB/c mice to C57BL/6 mice with or without CHBP treatment. A secondary syngeneic skin transplantation model, involving transplantation from C57BL/6 mice to C57BL/6 mice, was also created to act as a control. Skin graft rejection, CD19^+^ B cell infiltration in the skin allograft, the percentages of splenic plasma cells, germinal center (GC) B cells, and Tfh cells, the serum levels of donor specific antibodies (DSAs), and NF-*κ*B signaling in splenocytes were analyzed. Skin allograft survival was significantly prolonged in the CHBP group compared to the allogeneic group. CHBP treatment also significantly reduced the CD19^+^ B cell infiltration in the skin allograft, decreased the percentages of splenic plasma cells, GC B cells, and Tfh cells, and ameliorated the increase in the serum DSA level. At a molecular level, CHBP downregulated P100, RelB, and P52 in splenocytes. CHBP prolonged skin allograft survival by inhibiting AMR, which may be mediated by inhibition of NF-*κ*B signaling to suppress B cell immune responses, thereby decreasing the DSA level.

## Introduction

Antibody-mediated rejection (AMR) represents a major cause of allograft dysfunction and results in allograft failure in solid organ transplantation (1, 2). It is driven by circulating donor-specific antibodies (DSAs), which are mainly secreted by plasma cells differentiated from B cells (2, 3). The differentiation of B cells into plasma cells involves an ordered cascade of cellular events. After antigen stimulation, IgM^+^IgD^+^ naive B cells in secondary lymphoid follicles are activated and migrate to the T–B border, where B cells proliferate and interact with T follicular helper (Tfh) cells, completing the activation (4). Thereafter, some of the activated B cells enter the center of follicles to form germinal centers (GCs) (5). In GCs, Tfh cells instruct GC B cells to proliferate and undergo affinity maturation, class switching, and eventually, differentiation into long-lived plasma cells (6). As AMR, which is mediated by B cell immune responses, is an important contributor to allograft dysfunction and failure after organ transplantation and lacks effective therapies, there is an urgent need for novel therapies for AMR.

Cyclic helix B peptide (CHBP) is a novel erythropoietin (EPO)-derived peptide (7). It was synthesized by our group in 2014 and has a thioether-cyclized structure that is resistant to proteolytic degradation while exerting its tissue protective effects. CHBP achieves its effects by binding to tissue protective receptor (TPR) and does not induce the side effects or toxicity of EPO (7). Our previous studies revealed that CHBP restored renal function and/or attenuated pathological changes in a renal ischemia–reperfusion injury (IRI) model, an aristolochic acid-induced acute kidney injury model, a unilateral IRI-induced renal fibrosis model, and a unilateral ureter obstruction-induced renal fibrosis model (8–12). Additionally, the tissue protective effect has also been shown in an acute myocardial infarction model, in which CHBP ameliorated cardiac injury and improved cardiac function (13). Moreover, there has been a single study on the effect of CHBP on allograft rejection in a renal transplantation model, and it yielded positive results (14). Thus, CHBP may inhibit transplantation rejection. The effect of CHBP on AMR, as a type of transplantation rejection, was therefore investigated.

In this study, a secondary skin transplantation model was established to evaluate the effect of CHBP on skin allograft survival. We also investigated whether the mechanism of AMR inhibition involves inhibition of B cell immune responses.

## Materials and Methods

### Experimental Animals

The donor mice were 8-week-old male BALB/c or C57BL/6 mice. The recipient mice were 8-week-old male C57BL/6 mice. The mice were purchased from Shanghai SLAC Laboratory Animal Co., Ltd. They were bred in a specific-pathogen-free experimental animal room with a 12/12-h light/dark cycle. Before the experiments, the mice were left to acclimatize for 1 week, with free access to food and water. All animal procedures were performed in accordance with bioethics guidelines and were approved by the Bioethics Committee of Zhongshan Hospital, Fudan University, Shanghai, China.

### Skin Transplantation Model

Twenty-four recipient C57BL/6 mice were randomly assigned to four groups (n = 6 each): (1) syngeneic group (donors: C57BL/6 mice), (2) allogeneic group (donors: BALB/c mice), (3) low-dose CHBP (donors: BALB/c mice) group, and (4) high-dose CHBP (donors: BALB/c mice) group. The mice in the former two groups were injected with normal saline (NS) daily after the secondary transplantation. The mice in the latter two groups, *i.e.*, the low- and high-dose CHBP groups, were injected with 100 and 500 µg/kg CHBP daily, respectively, after the secondary transplantation.

To prepare the donor skin, both C57BL/6 and BALB/c mice were sacrificed by cervical dislocation. Skin was removed from the tails, placed in cold saline, and cut into 1.0 × 1.5 cm^2^ pieces. To prepare the recipient mice, C57BL/6 mice were anesthetized with intraperitoneal pentobarbital (0.1 g/kg). After being shaved and disinfected, a 1.0 × 1.5 cm^2^ graft bed was cut in the skin on the dorsal neck of each recipient mouse. Next, donor skin was placed into the graft bed and stitched with absorbable 6-0 sutures. The primary skin transplantation was performed on day −14, and a secondary transplantation was performed on day 0. Each graft site was wrapped with a bandage, and photographs were taken daily from day 5 after secondary transplantation with a digital camera for 19 days or until the graft was rejected completely.

After the secondary skin transplantation, hardened, sloughed off, or necrotic skin indicated immune rejection, while ruddy soft skin with hair indicated lack of immune rejection.

### Hematoxylin and Eosin Staining

Skin grafts from the recipient mice were harvested on day 10, fixed in 4% paraformaldehyde, dehydrated in ethanol, and embedded in paraffin. The skin tissue was then cut into 5-µm sections, deparaffinized in xylene, and rehydrated using a graded ethanol series before H&E staining. The structure and inflammatory cell infiltration of skin allografts (0: absent; 1: discrete; 2: moderately discrete; 3: moderate; 4: moderately severe; 5: severe) were observed and photographed under light microscopy.

### Immunohistochemical Staining

The paraffin-embedded skin sections were deparaffinized, rehydrated, and blocked with 3% hydrogen peroxide and then incubated at 37°C for 1 h with anti-CD19 antibody (1:1,600; Cell Signaling Technology, Inc., Danvers, MA, USA). Thereafter, the sections were washed with phosphate-buffered saline (PBS) and incubated at room temperature for 1 h with horseradish peroxidase (HRP)-conjugated secondary antibody (1:200; Abcam). The reaction was subsequently visualized with 3,3′-diaminobenzidine solution (Gene Tech Co., Ltd., Shanghai, China), and the sections were counterstained with hematoxylin. Semi-quantification of the IHC staining results was performed under a high-power field (400×).

### Flow Cytometry Analysis

To obtain spleen cell suspensions, spleens were harvested from recipient mice on day 10, milled gently in PBS supplemented with 1% fetal bovine serum using a 10-ml syringe, and forced through a 200-µm nylon mesh. Lymphocytes were isolated from the spleen cell suspensions using lymphocyte separation solution (Dakewe Biotech Co., Ltd., Shenzhen, China) according to the manufacturer’s instructions. Thereafter, the isolated lymphocytes were washed with PBS and incubated with the following fluorochrome-conjugated antibodies (all from BD Biosciences, New York, USA): FITC-GL7 (1:100; #553666), APC-B220 (1:100; #553092), PE-Fas (1:100; #555293), FITC-CD4 (1:100; #553651), APC-CXCR5 (1:100; #560615), PE-ICOS (1:100; #552146), FITC-CD19 (1:100; #553666), and APC-CD138 (1:100; #561705), and then assessed using a BD FACS Aria II Cell Sorter (BD Biosciences). The percentages of CD4^+^CXCR5^+^ICOS^+^ Tfh cells, GL7^+^B220^+^Fas^+^ GC B cells, and CD19^+^CD138^+^ plasma cells were analyzed using FlowJo software 6.0 (Tree Star Inc., Ashland, OR, USA).

### Circulating Donor-Specific Antibodies

The levels of circulating DSA-IgG and DA-IgM in recipient serum were assessed by flow cytometry on day 10 as previously described (15). Briefly, recipient serum was obtained by centrifugation at 2,000 g and 20°C for 10 min. Donor lymphocytes were isolated from spleen cell suspensions using lymphocyte separation solution (Dakewe Biotech Co., Ltd.). Thereafter, the recipient serum was incubated with the donor lymphocytes at 37°C for 30 min. After being washed with PBS, the lymphocytes were incubated with anti-mouse IgG (1:100; #405305; BD Biosciences) and anti-mouse IgM (1:100; #406509; BD Biosciences) at 4°C for 1 h. Subsequently, the cells were analyzed by flow cytometry, with the results represented as mean fluorescence intensity to reflect the individual serum DSA levels.

### Western Blot Analysis

Recipient splenic lymphocytes were obtained using lymphocyte separation solution (Dakewe Biotech Co., Ltd.) according to the manufacturer’s instructions. The lymphocytes were homogenized in ice-cold radioimmunoprecipitation assay lysis buffer containing phenylmethylsulfonyl fluoride (PMSF) and phosphatase inhibitor. Lysates were centrifuged at 12,000 g and 4°C for 25 min, and the supernatant was subsequently collected. Total proteins were quantified by bicinchoninic acid assay. Thereafter, 20 µg proteins were separated *via* 10% sodium dodecyl sulfate-polyacrylamide gel electrophoresis and transferred onto a polyvinylidene difluoride membrane. After being blocked with 5% milk for 1 h, the membrane was incubated at 4°C overnight with the following primary antibodies (all from Cell Signaling Technology, Inc.): anti-P100/P52 (1:1,000; #4882), anti-RelB (1:1,000; #10544), and anti-β-actin (1:1,000; #3700). The membranes were washed using Tris-buffered saline with Tween-20 and incubated with horseradish peroxidase-conjugated goat anti-rabbit secondary antibody (1:5,000; Abcam) at room temperature for 1 h. The proteins were visualized using an enhanced chemiluminescence system (Thermo Fisher Scientific, Rockford, IL, USA). Band intensity was analyzed using ImageJ software and normalized to the value of *β*-actin.

### Statistical Analysis

Data are presented as mean ± standard deviation (SD). SPSS 19.0 software (IBM Corp., Armonk, NY, USA) was used for statistical analysis of data among the three groups, using one-way analysis of variance followed by Bonferroni’s correction. Differences in animal survival were analyzed using the log-rank test (with Kaplan–Meier survival curves). P <0.05 was considered statistically significant.

## Results

### CHBP Prolonged Skin Allograft Survival

**Figure 1:**
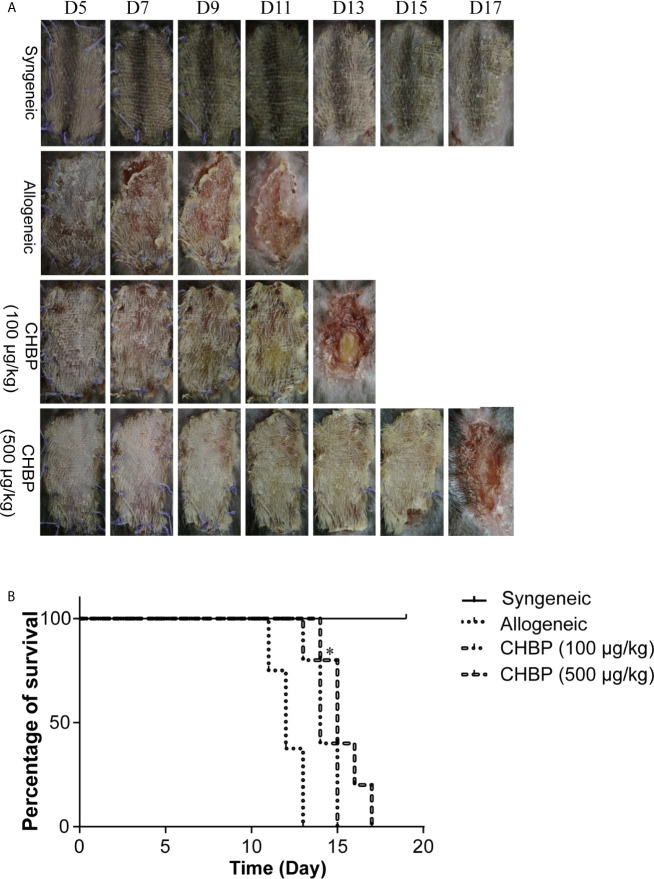
CHBP prolonged skin allograft survival. **(A)** Photographs of skin allografts at various time points after secondary transplantation in each group. **(B)** Kaplan–Meier survival curves of mice in each group by day of post-transplantation. *P < 0.05: This P value is compared between the allogeneic group and the CHBP (500 µg/kg) group.

We first investigated the effect of CHBP on skin allograft survival. Daily treatment with NS or CHBP was started after completion of the secondary skin transplantation. As shown in **Figure 1A**, the syngeneic group showed normal skin growth without hardening, incrustation, necrosis, or rejection. In contrast, the allogeneic group exhibited obvious rejection, starting from day 5, after the bandage was removed (which occurred on day 5), including progressive loss of hair, hardening, dermal necrosis, and scab formation. The first rejection was observed in the low-dose CHBP group (100 µg/kg) on day 6 and in the high-dose CHBP group (500 µg/kg) on day 9.

Consistent with skin allograft observations, the survival analysis showed that the syngeneic group displayed no rejection of skin allografts. In contrast, rejection in the allogeneic group started on day 11, and all the skin allografts were rejected by day 13. Additionally, rejection in the low-dose CHBP group started on day 12 and was completed on day 15, while rejection in the high-dose CHBP group started on day 14 and was completed on day 17 (**Figure 1B**). These results suggested that CHBP prolonged skin allograft survival, which was especially obvious in the high-dose group.

### CHBP Decreased Inflammatory Cell Infiltration in the Skin Allograft

**Figure 2:**
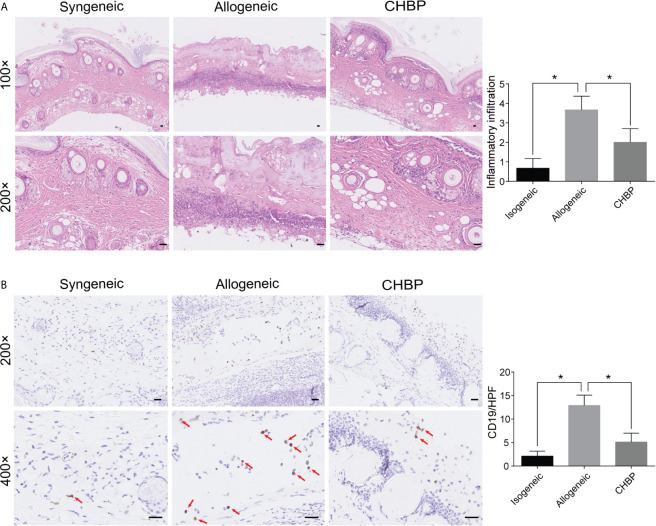
CHBP decreased the inflammatory cell infiltration and CD19+ B cell infiltration in skin allografts. **(A)** Representative images of H&E staining in the syngeneic, allogeneic, and CHBP groups and semi-quantification of inflammatory cell infiltration in the three groups. **(B)** Representative images of immunohistochemical staining (IHC) for CD19 in the syngeneic, allogeneic, and CHBP groups and semi-quantification of IHC staining in the three groups. *P < 0.05.

Based on the previous result, we used a dose of 500 µg/kg CHBP for the subsequent experiments. Histological examination of skin allografts in the syngeneic group on day 10 after secondary transplantation showed that the skin structure had normal morphology and low inflammatory cell infiltration in the dermis and subcutaneous tissue (**Figure 2A**). In contrast, obvious skin peeling and severe inflammatory cell infiltration was observed in the allogeneic group. CHBP improved the skin structure and led to a significant moderate decrease in the inflammatory cell infiltration.

### CHBP Decreased CD19^+^ B Cell Infiltration in the Skin Allograft

To investigate whether the CHBP-induced decrease in inflammatory cell infiltration involved B cells, CD19^+^ B cells in skin allografts were subjected to immunochemistry staining. As shown in **Figure 2B**, the CD19^+^ B cell infiltration was significantly increased in the allogeneic group compared to the syngeneic group. CHBP significantly decreased CD19^+^ B cell infiltration compared to the level in the allogeneic group, implying that the prolonged skin allograft survival may be due to decreased CD19^+^ B cell infiltration.

### CHBP Decreased the Percentage of Plasma Cells in the Spleen

**Figure 3:**
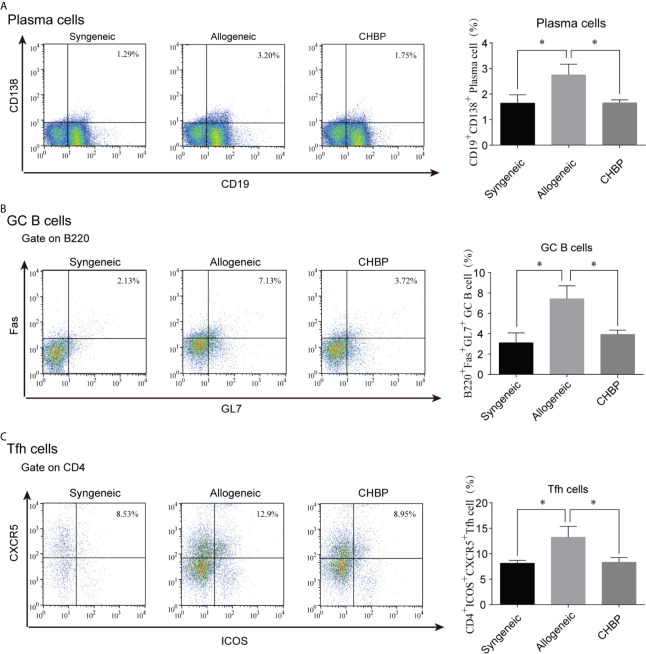
CHBP decreased the percentages of plasma cells, GC B cells, and Tfh cells in the spleen. Percentages of **(A)** CD19^+^CD138^+^ plasma cells, **(B)** GL7^+^B220^+^Fas^+^GC B cells, and **(C)** CD4^+^CXCR5^+^ICOS^+^ Tfh cells in the spleen in each group. *P < 0.05.

Plasma cells are important factors in organ rejection. To confirm whether plasma cells were altered (among the CD19^+^ B cells), the percentage of plasma cells in the spleen of recipient mice was analyzed. As shown in **Figure 3A**, flow cytometry revealed that CD19^+^CD138^+^ plasma cells in the spleen 10 days after secondary transplantation were significantly increased in the allogeneic group compared to the syngeneic group. However, CHBP ameliorated the increase of plasma cells, with a significantly lower level compared to the level in the allogeneic group. This shows that CHBP decreased the percentage of plasma cells in recipient mice.

### CHBP Decreased the Percentages of GC B Cells and Tfh Cells in the Spleen

Plasma cells are differentiated from GC B cells after being instructed by Tfh cells. The percentages of both GL7^+^B220^+^Fas^+^ GC B cells and CD4^+^CXCR5^+^ICOS^+^ Tfh cells were assessed by flow cytometry. The results revealed that the percentages of GC B cells and Tfh cells were significantly increased in the allogeneic group compared to the syngeneic group. CHBP significantly decreased the percentages of these cells compared to the levels in the allogeneic group (**Figures 3B, C**), suggesting that CHBP may reduce the differentiation of GC B cells into plasma cells by decreasing the percentages of Tfh cells and GC B cells.

### CHBP Decreased the DSA Levels in Recipient Mice

**Figure 4:**
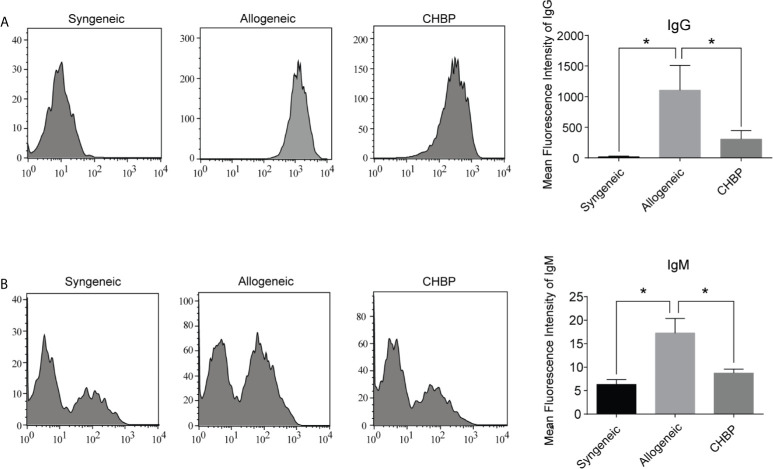
CHBP decreased donor-specific antibodies (DSAs) in recipient mice. **(A)** DSA-IgG and **(B)** DSA-IgM level in recipient serum in each group were detected by flow cytometry and expressed as mean fluorescence intensity (MFI). *P < 0.05.

DSAs are secreted by plasma cells. To assess whether CHBP affects the secretion of DSAs, the DSA-IgG and DSA-IgM levels in the serum of recipient mice were assessed 10 days after secondary skin transplantation. As shown in **Figure 4**, the levels of both DSA-IgG and DSA-IgM were significantly elevated in the allogeneic group compared to the syngeneic group. CHBP ameliorated these increases, with significantly lower levels compared to the levels in the allogeneic group, implying that CHBP may decrease the percentage of Tfh cells and GC B cells to reduce the differentiation of GC B cells into plasma cells, thereby decreasing the DSA levels.

### CHBP Inhibited the NF-*κ*B Signaling Pathway

**Figure 5:**
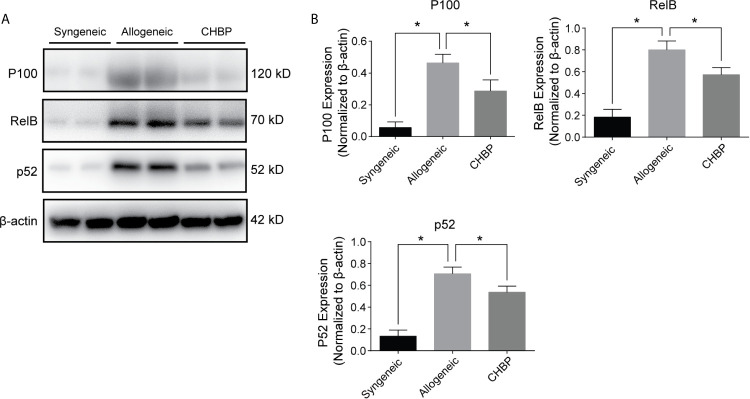
CHBP inhibited NF-*κ*B signaling pathway. **(A)** Representative western blot images of P100, RelB, P52, and *β*-actin in the syngeneic, allogeneic, and CHBP groups. **(B)** Ratios of P100, RelB, and P52 to *β*-actin. *P < 0.05.

The NF-*κ*B signaling pathway is reported to be involved in B cell immune responses. To investigate whether the NF-*κ*B pathway is involved in the inhibition of B cell immune responses by CHBP, we used western blotting to evaluate the protein expression of P100, RelB, and P52 in splenic lymphocytes. As shown in **Figure 5**, the NF-*κ*B signaling pathway was activated in the splenic lymphocytes in the allogeneic group, as evidenced by significantly upregulated P100, RelB and P52 compared to the levels in the syngeneic group. CHBP obviously downregulated these proteins compared to the levels in the allogeneic group, suggesting that CHBP may inhibit the NF-*κ*B signaling pathway and thereby inhibit B cell immune responses and decrease the DSA levels, thus reducing the occurrence of AMR.

## Discussion

CHBP is a newly synthesized peptide derived from EPO. Our previous studies indicated that CHBP protects against various conditions, including renal IRI, acute kidney injury, renal fibrosis, and acute myocardial infarction (8–13). Additionally, research has indicated that CHBP can ameliorate acute renal allograft rejection (14). Herein, the effect of CHBP on AMR was investigated. We found that treatment with CHBP prolonged skin allograft survival. The mechanism may involve inhibition of the NF-*κ*B signaling pathway in order to inhibit B cell immune responses, thereby decreasing the DSA level and the occurrence of AMR.

AMR is mediated by immune responses involving B cells, which are one of the major cellular components that play vital roles in allograft rejection. AMR is the most vexing barrier to organ transplantation. The contributions of B cells during allograft rejection are reported to include serving as antigen-presenting cells to regulate the immune response, promoting CD4^+^ T cell expansion and memory formation, and differentiating into plasma cells to secrete antibodies (16). GCs are specialized structures that conventionally form within secondary lymphoid organs, such as the spleen. In GCs, B cells undergo proliferation, hypermutation, selection, and differentiation, generating plasma cells (17, 18). The GC response arises from the interactions between B and Tfh cells. Tfh cells are a specialized subset of CD4^+^ T helper cells, located in GCs to instruct GC B cells to proliferate and differentiate into plasma cells (19). Multiple studies have revealed that Tfh cells play an important role in B cell activation and antibody production. Chen et al. revealed that mice immunized with IL-7-overexpressing recombinant canine distemper virus (rCDV) exhibited significantly increased Tfh cell generation, GC formation, GC B and plasma cell generation, and antibody production (20). Furthermore, Elsner et al. reported that using IL-12 to block Tfh cell differentiation contributed to GC suppression, inhibiting humoral immunity (21), suggesting that inhibition of Tfh cell generation inhibits B cell immune responses and antibody production. In this study, we discovered that the percentages of Tfh cells and GC B cells were significantly elevated in the allogeneic group compared to the syngeneic group, while CHBP significantly decreased the percentages of these cells compared to the levels in the allogeneic group. Similar changes in the percentages of plasma cells were also observed, suggesting that the CHBP-induced prolonged skin allograft survival may be achieved by decreasing the percentages of Tfh cells and GC B cells, thus reducing the differentiation of GC B cells into plasma cells.

The differentiation of B cells into plasma cells to secrete DSAs is a critical component of the development of AMR and a major risk factor for allograft rejection (22–24). Given that the percentage of plasma cells decreased, the DSA levels were subsequently assessed. Consistent with the finding that CHBP significantly decreased the percentage of plasma cells compared to the level in the allogeneic group, corresponding significant decreases in the DSA-IgG and DSA-IgM levels were detected in the CHBP group compared to the allogeneic group. This implies that CHBP decreases the percentages of Tfh and GC B cells to reduce the differentiation of GC B cells into plasma cells; this, in turn, lowers the DSA levels, thus prolonging skin allograft survival.

The NF-*κ*B signaling pathway is commonly regarded as being associated with cellular proliferation and plays an important role in the regulation of both innate and adaptive immunity (18, 25). B cells are one of the most critical components of innate and adaptive immunity, and B cell survival, apoptosis, proliferation, and class switch recombination are dependent on appropriate regulation of the NF-*κ*B pathway (19, 25). Evidence from multiple studies has revealed that mice with constitutive activation of the NF-*κ*B pathway exhibit autoimmunity, lymphomagenesis, and B cell hyperplasia (26–28), indicating the potential effects of NF-κB pathway activation on B cells. Another study indicated that conditional deletion of the transcription factor Relb in GC B cells led to the collapse of established GCs after day 7 of the GC response (29). In addition, knockout of the central component of the non-canonical NF-*κ*B signaling pathway, NF-*κ*B-inducing kinase (NIK), resulted in decreased Tfh cell generation (30), suggesting that the non-canonical NF-*κ*B pathway may promote the development of Tfh cells and the GC response.

The NF-*κ*B cascade is known to comprise two pathways, the canonical and non-canonical NF-*κ*B pathways. The canonical NF-*κ*B pathway results in the nuclear translocation of Rel, p65, and p50, and it is mainly involved in broad inflammatory responses in various cell types of the lymphoid system. The non-canonical NF-*κ*B pathway leads to the nuclear translocation of RelB and P52, and it exhibits specific functions related to B cell biology (29, 31). In this study, non-canonical pathway signaling was assessed, and CHBP obviously downregulated P100, RelB, and P52, suggesting that CHBP may inhibit the NF-*κ*B signaling pathway and thereby inhibit B cell immune responses, decrease the DSA levels, reduce the occurrence of AMR, and prolong skin allograft survival.

The main limitation of this study is that the NF-*κ*B signaling pathway was assessed in splenic lymphocytes but not specifically in T and B cells. Compared to detection in splenic lymphocytes, specific detection in T and B cells would provide stronger evidence to explain the reduction in GC B and Tfh cells. Additionally, we only revealed that CHBP inhibited the occurrence of AMR in a secondary skin rejection model. The role of CHBP in AMR in other organ transplantation models, such as renal and lung transplantation, needs to be further explored.

In conclusion, this study revealed that CHBP prolonged skin allograft survival in mice. The mechanism may involve the inhibition of the NF-*κ*B signaling pathway in order to inhibit B cell immune responses, thereby decreasing the DSA level and the occurrence of AMR.

## Data Availability Statement

The original contributions presented in the study are included in the article/supplementary material. Further inquiries can be directed to the corresponding authors.

## Ethics Statement

The animal study was reviewed and approved by the Ethics Committee of Zhongshan Hospital, Fudan University.

## Author Contributions

DZ, CY, and RR conceived the study and participated in its design. LZ, XW, LH, WG, WZ, and XZ carried out the experiments. CH and LZ analyzed the data and drafted the manuscript. RR, CY, and DZ obtained the funding. All authors contributed to the article and approved the submitted version.

## Funding

This study was supported by National Natural Science Foundation of China (81500569 to DZ, 81770747 to RR, 81770746 to CY, 81800659 to XW), National Key R&D Program of China (2018YFA0107501 to RR, 2018YFA0107502 to CY), Shanghai Rising-Star Program (19QA1406300 to CY), Medical and Health Talents Training Plan for the Excellent Youth of Shanghai Municipal (2018YQ50 to CY), 2019 Shanghai Youth Talent Development Program (to CY), and Fujian Provincial Health and Health Career Training Project for Young and Middle-aged Talents (2020GGB058 to DZ).

## Conflict of Interest

The authors declare that the research was conducted in the absence of any commercial or financial relationships that could be construed as a potential conflict of interest.
